# Left bundle pacing in transposition of the great arteries with previous atrial redirection operation

**DOI:** 10.1016/j.hrcr.2021.12.001

**Published:** 2021-12-07

**Authors:** Matthew O’Connor, S. Yen Ho, Karen P. McCarthy, Michael Gatzoulis, Tom Wong

**Affiliations:** ∗ Department of Electrophysiology, Royal Brompton Hospital, London, United Kingdom; † Adult Congenital Heart Centre, Royal Brompton Hospital, London, United Kingdom; ‡ Cardiac Morphology Unit, Royal Brompton Hospital, London, United Kingdom

**Keywords:** Transposition of the great arteries, Left bundle branch pacing, ACHD, Conduction system, Complete heart block, Mustard operation, atrial switch

## Introduction

Transposition of the great arteries (TGA) is a congenital heart defect characterized by concordant atrioventricular (AV) and discordant ventriculoarterial connections leading to 2 parallel circulatory systems. There are a number of operations that have been performed over the years to surgically palliate this parallel circulation; currently the arterial switch operation is the standard of care. There are occasions where the arterial switch operation is not feasible (for example, owing to hostile coronary anatomy) and alternative surgical approaches such as the more historical Mustard/Senning operations must be used. These atrial switch operations were first successfully performed in 1952 and there continues to be a large population of patients with such anatomy.^1^ In short, the Mustard operation consists of the superior vena cava (SVC) and inferior vena cava being rerouted via surgical baffle to the vestibule of the left atrium, with the subsequent redirection of systemic venous blood to the subpulmonary left ventricle (LV). The remaining pulmonary venous component of the left atrium is redirected rightward through the right atrium into the systemic right ventricle (RV).

A significant proportion of patients who have undergone an atrial switch operation will develop complete AV block and require pacing.^2^ Furthermore, up to 25% will develop systemic RV failure that is precipitated or exacerbated by chronic ventricular pacing.^3^^,^^4^ Cardiac resynchronization therapy (CRT) has been shown to prevent or improve LV dysfunction in appropriately selected patients with normal cardiac anatomy^5^ and its use has extended with evidence of symptomatic benefit into the adult congenital heart disease population. Importantly, the coronary sinus is inaccessible via a venous approach in 50% of atrial switch patients, as the ostium may drain into the pulmonary venous atrium postoperatively.

His-bundle pacing has gained traction as an alternative to CRT with a rapidly emerging evidence base and inclusion in major guidelines.^6^^,^^7^ Elevated thresholds have been reported during follow-up of His bundle leads; the etiology of this finding is not established, but is potentially due to lead microdislodgement or direct injury to the His bundle. Left bundle pacing (whereby the pacing lead is tunneled through the ventricular septum to the left bundle area) has developed as a viable alternative.^8^ Owing to the position deep in the septum, the issues of both microdislodgement and direct His bundle injury are addressed. It has also been employed in the adult congenital heart disease (ACHD) population, such as in congenitally corrected TGA.^9^ However, the implant complexity increases in TGA, particularly as implant tools—namely the C315 sheath (Medtronic, Minneapolis, MN)—are designed to implant a lead into a septum in the usual anatomical arrangement, where the ventricular septum is located posteriorly within the right ventricle instead of the anterior angulation required when implanting via a subpulmonary LV. We describe the technique of left bundle pacing in a patient with TGA and a previous atrial switch operation guided by electroanatomical mapping.

## Case report

**Figure 1:**
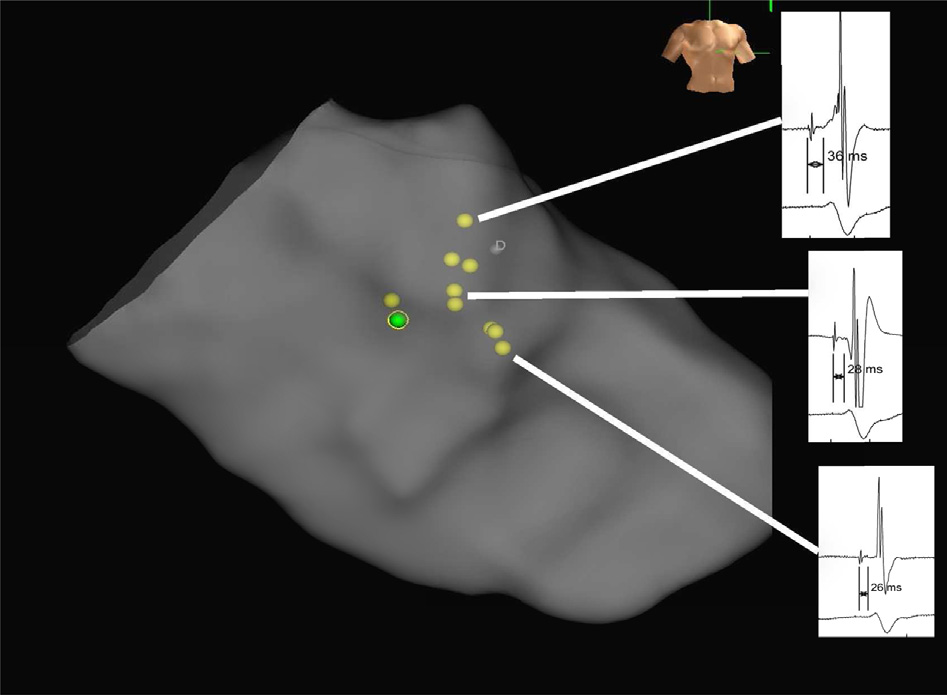
Electroanatomical map (RAO) with conduction tissue tagged (*yellow*) and final lead position (*green*) highlighted. Electrograms from specific locations demonstrate progressive shortening of the “HV interval” with more distal conduction tissue sampling.

A 61-year-old man with a history of TGA and an atrial switch operation (Mustard) at the age of 12 who had been lost to follow-up for 18 years presented to an outside center with breathlessness, fatigue, and prominent peripheral venous dilation. A 12-lead electrocardiogram demonstrated complete heart block with underlying sinus rhythm, QRS duration 142 ms with a right bundloid morphology (Figure 1). Holter monitoring revealed high-grade AV block with >5-second pauses during waking hours. Cardiac magnetic resonance imaging demonstrated stenosis of both SVC and inferior vena cava baffles with mildly impaired systemic RV function (ejection fraction 56%) and that the coronary sinus drained into the pulmonary venous atrium. The baffles were percutaneously stented with 2 stents (CP Stent, 39 mm; NuMED Inc, Hopkinton, NY) via the right internal jugular vein and the right femoral vein, with excellent angiographic and hemodynamic result.

The patient then attended the electrophysiology laboratory 2 days later for implantation of a dual-chamber left bundle area pacemaker under general anesthesia. Owing to the angulation of the baffles, inferior access for mapping was considered unfavorable and access was via the right basilic vein. The basilic vein measured 29 mm in diameter and accepted a 6F and an 8F sheath placed via the modified Seldinger technique under ultrasound guidance. A fixed-curve quadripolar catheter (St Jude Medical, St Paul, MN) was placed in the subpulmonary LV apex to provide back-up pacing.

The 3D electroanatomical mapping system, EnSite Precision with the Advisor HD grid mapping catheter (Abbott Inc, Abbott Park, IL), was used to map and tag conduction system potentials within the subpulmonary LV (Figure 1).

**Figure 2:**
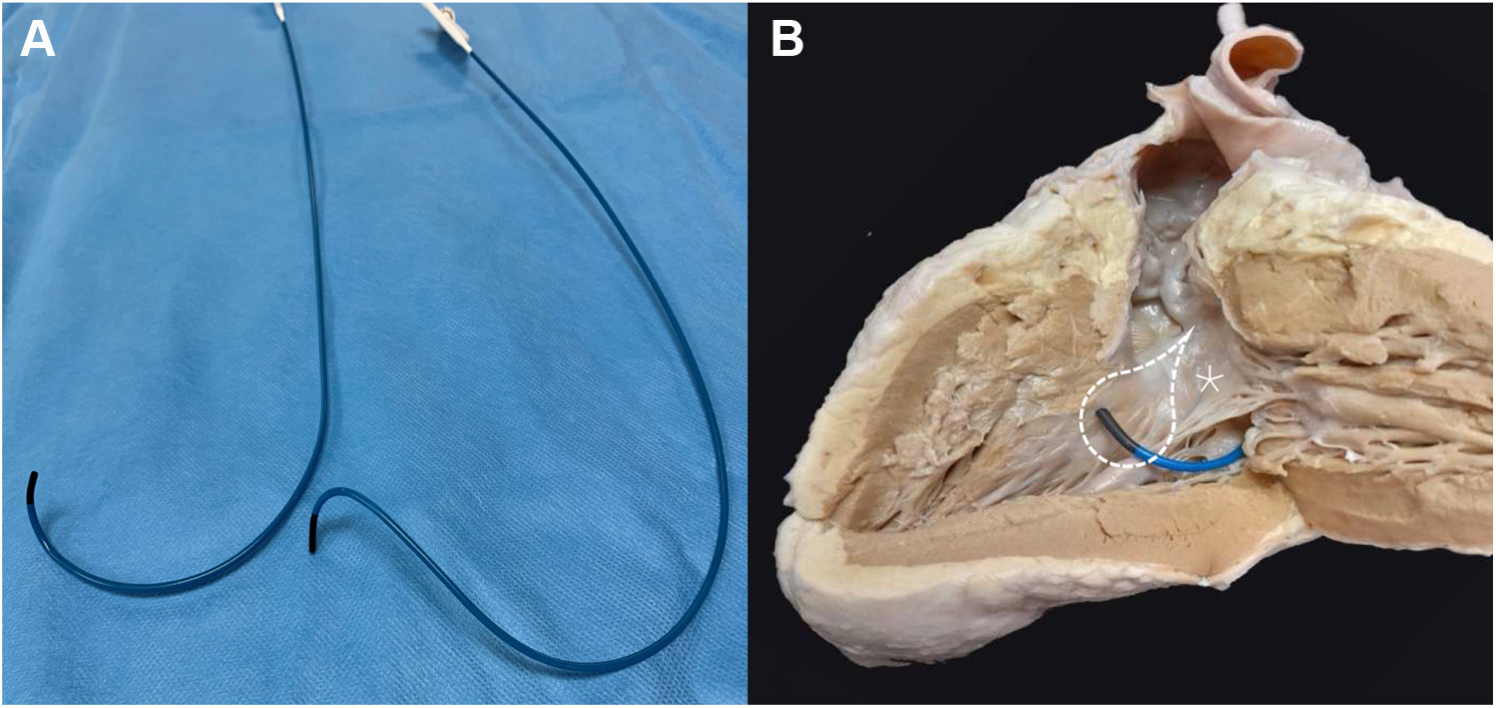
**A:** An original C315 sheath (Medtronic, Minneapolis, MN) (left) and the reshaped sheath (right) to adapt for the left bundle pacing in transposition of the great arteries with previous atrial redirection operation. Mustard anatomy with a deep primary curve under the mitral valve and an anterior or right-hand secondary curve to the septum. **B:** The reshaped C315 sheath in an explanted Mustard heart with the subpulmonary left ventricular free wall reflected to demonstrate the different shape required to reach the left bundle. Dashed lines outline the approximate location of the proximal left bundle conduction system. marks the anterior mitral valve leaflet.

The HD grid has a relatively large footprint (13 × 13 mm) but can be compressed to fit through an 8.5F sheath. To facilitate direct delivery of the HD grid into the subpulmonary LV, and avoid damage to the peripheral veins or the newly implanted (and thus nonendothelialized) SVC stent, the 8F short sheath was exchanged for an 8.5F SLO sheath (St Jude Medical), which was advanced over the guidewire, leaving the tip of the sheath just beyond the SVC stent. In addition to the 3D electroanatomical map, fluoroscopic images of the HD grid, positioned over conduction system potentials, were taken in orthogonal views (left anterior oblique 30 and right anterior oblique 30 degrees) to act as a second reference. Once a suitable left bundle target was identified, a standard left-sided infraclavicular incision was made and axillary vein access obtained under ultrasound guidance. A C135 sheath (Medtronic) was manually reshaped (Supplemental Video 1) to the atrial switch anatomy of the patient by reversing the direction of the primary curve and augmenting the secondary curve (Figure 2). With the sheath modified in this fashion, clockwise torque by the operator, once in the LV, placed the tip of the sheath against the interventricular septum.

**Figure 3:**
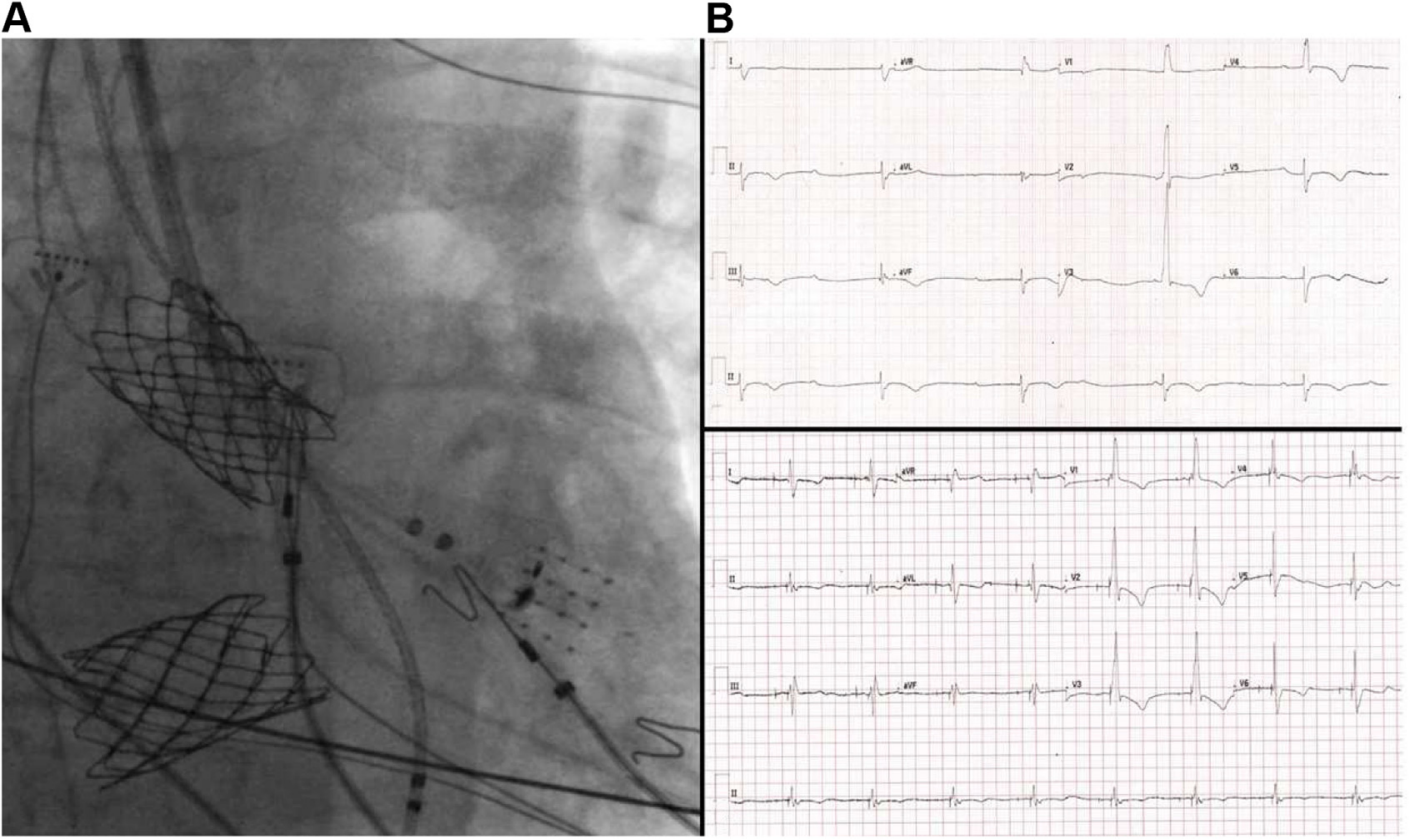
**A:** Fluoroscopic image demonstrating superior vena cava and inferior vena cava baffles and final lead position with reference to the HD grid catheter. **B:** Presenting electrocardiogram (ECG) demonstrating sinus rhythm, complete AV block, and a narrow complex escape rhythm. **C:** Final paced ECG demonstrating left bundle capture with almost identical QRS morphology to the intrinsic QRS.

The distal pole of a 3830 SelectSecure lead (Medtronic) was connected in a unipolar fashion to the mapping system to facilitate visualization of the lead tip, allowing it to be guided into the conduction system via the modified C315 sheath. The position was confirmed fluoroscopically before deploying the lead (Figure 3).

In contrast to the traditional placement of the pacing lead in left bundle area (where both electrodes are located within the septum), the lead was tunneled so that just the distal electrode was in the septum owing to the relatively superficial location of the left bundle system when approached from the morphological left ventricle. A second lead was deployed in the left atrial appendage.

Electrical parameters were excellent, with left bundle capture threshold at 1.0 V @ 1.0 ms. The resultant QRS duration (142 ms) and morphology were almost identical to that of the intrinsic QRS.

## Discussion

Systemic RV dysfunction, frequently exacerbated by LV apical pacing, is an important cause of morbidity and mortality in this population.^4^ It is accepted that CRT is beneficial and possible, but requires an epicardial or hybrid transvenous/epicardial approach. Providing physiological pacing via a completely transvenous approach avoids the additional risk of cardiothoracic surgery. Cano and colleagues^10^ have demonstrated feasibility of conduction system pacing in ACHD; our use of visualization of the pacing lead through the electroanatomical mapping system allows for directly guided lead placement into the left bundle.

In TGA the conduction system is located in the traditional anatomical location within the ventricles in a similar distribution to that in usual cardiac anatomy. Complexity arises from the need to deliver the left bundle lead from the LV when the implant sheaths are designed for an RV approach to the septum. The C135 sheath is designed for delivery of a 3830 lead onto the His bundle, and more recently has been used for implantation in the septum for left bundle area pacing. It has a primary curve designed to angle the sheath rightward (when viewed in an anteroposterior projection) through the right atrium and tricuspid valve, and a secondary curve at the distal tip with a 90-degree offset allowing for septal (posterior) angulation. In patients with TGA and atrial switch, the subpulmonary LV is posterior relative to the systemic RV; without modification, the C315 sheath would be directed toward the posterolateral LV wall (away from the septal left bundle system). To address this, we reshaped the primary curve of the C315 sheath manually such that it was reversed by 180 degrees. The catheter can then be “flipped”—thus retaining the primary angulation, but reversing the secondary curve to face anteriorly toward the septum (Supplemental Video 1). It is important to perform this maneuver with the dilator in situ to prevent deformation of the lumen of the catheter, which may subsequently impede torque transmission during lead deployment.

Adding further complexity to the desired shape is that the anterior leaflet of the mitral valve opens toward the septum and must be circumnavigated—necessitating a much deeper primary curve and sharper distal curve. This is not an issue on the right side, as the lead deployment location approximates the raphe between the septal and posterior leaflets of the tricuspid valve. Reshaping the C315 sheath is a well-known and well-used technique to achieve His bundle and left bundle pacing in usual cardiac anatomy, but the adjustments required to the sheath shape are minimal in these instances.^11^ In our case the adjustment was more radical, with complete reversal of the primary curve; however, we found the sheath reshaping to be well tolerated by the sheath and the new shape retained for the duration of the implant.

Left bundle pacing provides synchronous activation of the left ventricle, but the long-term effects of the resultant right bundle branch block (RBBB) have not been studied. Spontaneous, lone RBBB has no adverse effect in the general population, but in patients with a systemic RV the effect of the resultant RBBB is unknown.^12^ Subpulmonary LV dysfunction is associated with an increased incidence of clinical heart failure and biventricular CRT has been shown to improve systemic RV function.^13^^,^^14^ There is a need for collaboration between groups to establish if conduction system pacing has a similar beneficial effect and what role it has in the future of ACHD pacing.^15^

## Conclusion

Conduction system pacing in atrial switch TGA patients is feasible, maintains intrinsic ventricular depolarization, and may provide improved long-term outcomes regarding systemic RV function. Implant tools currently available require substantive adaptation to adjust for patient-specific anatomy, and the use of electroanatomical mapping is helpful to guide the implant. Given the heterogeneity of anatomy in complex ACHD patients, further study is warranted to confirm reproducibility of this technique and correlate with physiological and clinical outcomes.

**Key Teaching Points**

• Left bundle pacing in complex adult congenital heart disease anatomy such as transposition of great arteries post-atrial switch is feasible with potential benefits.
• Left bundle pacing can be facilitated by the use of 3D electroanatomical mapping with visualization of the pacing lead.
• The Medtronic C315 sheath can be extensively re-shaped and adapted to suit complex anatomies; doing so with the dilator in situ is important to prevent damage to the sheath.

## References

[1] Mustard W., Chute A., Keith J., Sirek A., Rowe R., Vlad P. (1954). A surgical approach to transposition of the great vessels with extracorporeal circuit. Surgery.

[2] Oechslin E., Jenni R. (2000). 40 years after the first atrial switch procedure in patients with transposition of the great arteries: long-term results in Toronto and Zurich. Thorac Cardiovasc Surg.

[3] Piran S., Veldtman G., Siu S., Webb G.D., Liu P.P. (2002). Heart failure and ventricular dysfunction in patients with single or systemic right ventricles. Circulation.

[4] Yeo W.T., Jarman J.W.E., Li W., Gatzoulis M.A., Wong T. (2014). Adverse impact of chronic subpulmonary left ventricular pacing on systemic right ventricular function in patients with congenitally corrected transposition of the great arteries. Int J Cardiol.

[5] Brignole M., Auricchio A., Baron-Esquivias G. (2013). 2013 ESC Guidelines on cardiac pacing and cardiac resynchronization therapy. Europace.

[6] Kusumoto F.M., Schoenfeld M.H., Barrett C. (2019). 2018 ACC/AHA/HRS Guideline on the Evaluation and Management of Patients With Bradycardia and Cardiac Conduction Delay: A Report of the American College of Cardiology/American Heart Association Task Force on Clinical Practice Guidelines and the Heart Rhythm Society. Circulation.

[7] Glikson M., Nielsen J.C., Kronborg M.B. (2021). 2021 ESC Guidelines on cardiac pacing and cardiac resynchronization therapy. Europace.

[8] Wu S., Su L., Vijayaraman P. (2021). Left bundle branch pacing for cardiac resynchronization therapy: nonrandomized on-treatment comparison with His bundle pacing and biventricular pacing. Can J Cardiol.

[9] Moore J.P., Gallotti R., Shannon K.M. (2020). Permanent conduction system pacing for congenitally corrected transposition of the great arteries: a Pediatric and Congenital Electrophysiology Society (PACES)/International Society for Adult Congenital Heart Disease (ISACHD) Collaborative Study. Heart Rhythm.

[10] Cano Ó., Dandamudi G., Schaller R.D. (2021). Safety and feasibility of conduction system pacing in patients with congenital heart disease. J Cardiovasc Electrophysiol.

[11] Vijayaraman P., Ellenbogen K.A. (2018). Approach to permanent His bundle pacing in challenging implants. Heart Rhythm.

[12] Zhang Z.-M., Rautaharju P.M., Prineas R.J., Loehr L., Rosamond W., Soliman E.Z. (2015). Ventricular conduction defects and the risk of incident heart failure in the Atherosclerosis Risk in Communities (ARIC) Study. J Card Fail.

[13] Surkova E., Segura T., Dimopoulos K. (2021). Systolic dysfunction of the subpulmonary left ventricle is associated with the severity of heart failure in patients with a systemic right ventricle. Int J Cardiol.

[14] Janousek J., Tomek V., Chaloupecký V.A. (2004). Cardiac resynchronization therapy: a novel adjunct to the treatment and prevention of systemic right ventricular failure. J Am Coll Cardiol.

[15] O’Connor M., Gatzoulis M., Wong T. (2021). Conduction system pacing in adults with congenital heart disease. Int J Cardiol Congenit Heart Dis.

